# The Sustainability Narrative: A Multi Study Using Event Studies to Analyse the American Energy Companies Shareholder’s Reaction to Sustainability News

**DOI:** 10.3390/ijerph192315489

**Published:** 2022-11-22

**Authors:** Alberto Barroso Del Toro, Laura Vivas Crisol, Xavier Tort-Martorell

**Affiliations:** 1 Department of Statistic and Operation Research, Universitat Politecnica de Catalunya, Jordi de Girona 31, 08034 Barcelona, Spain; 2 Independent Researcher, 221 85 Lund, Sweden

**Keywords:** sustainability news, financial markets, event study, GDELT, United States, greenwashing

## Abstract

This study investigates how shareholders of leading US energy companies value sustainability narratives. Leveraging the Global Database of Events (GDELT) from 2017 to 2019, 207,386 news items were extracted, 4101 event studies were performed, 3393 cumulative average abnormal returns (CAAR) were analysed, and 708 Abnormal volatilities (AV) were analysed. The magnitude of the analysis and further segmentation of the viral news by tone, type of energy, and environmental consequence help us to understand shareholders’ investment decisions and narrative. We proved that the sustainability narrative has a significant impact on shareholder value. There is a clear negative bias on sustainability news, impacting negatively on the market. More importantly, we’ve identified positive news about fossil fuels impacting the market more than positive renewable energy news. These results provide empirical evidence for the case of greenwashing in businesses. There must be a common shareholder’s narrative to penalise and reduce incentives for highly polluting investments to push forward an effective ecological transition. These results provide an objective for regulators to develop further regulations and incentives to fight against false sustainability news.

## 1. Introduction

The environmental, social and governance (ESG) concept has gained momentum over the last decade. In addition to citizens, media, and governments, global pressure has compelled several companies to establish new corporate objectives that are more respectful to the current environment and to adopt sustainable corporate practices [1,2,3,4,5].

In the past, commitments to the environment were seen by investors as merely an expense or an unproductive cost [6,7]. However, at present, the message that is being received is that the greater the commitment to ESG, the greater the long-term profitability [8,9,10,11,12,13,14].

Therefore, shareholders’ interest in companies committed to ESG has been increasing [15,16,17,18], not just because of greater profitability, but because of an ecological and social conscience [19,20,21]. Globally, sustainable investment assets increased by 34% from 2016 to 2018, reaching $30.7 trillion [22]. Consequently, the incentives to companies to be or to appear to be sustainable is increasing [14].

However, in the United States (US)—the world’s most active stock market—investments in ESG are lower than in Europe. In 2018, the US had $11.9 trillion invested in sustainable assets, accounting for only 25% of the total; meanwhile, European investments amounted to $14 trillion, accounting for 49% [23]. This difference in investment proportion is due to the significant dependence on fossil fuels in the American market—80% of the energy consumed comes from oil, coal, and natural gas, all of which are fossil fuels [24]. Another influential factor has been US regulations on sustainability, which moved into a phase of disinterest and even discouraging positions under the Trump administration.

However, this trend is changing with the new administration, with the return to the Paris agreement and the increasing presence of ESG companies in the stock market. Approximately eight in ten US investors are interested in sustainable investments, with half of them investing in at least one sustainable asset [22]. However, whilst more companies and investors than ever are claiming to be sustainable, CO_2_ emissions have been rising unabated [25]. This indicates that, first, the sustainability narrative is not aligned with practice, and second, there are clear incentives for greenwashing by companies.

Consequently, given the relevance that sustainability has acquired for investors and the unprecedented access to information that we currently have, it is highly probable that investors will react to sustainability news.

The motivation of this work is to understand the sustainability narrative of the US energy companies’ shareholders by exploring the following questions: Do shareholders follow sustainability news? Do they respond to negative sustainability news more than to positive or neutral? What type of energy makes the market more volatile? Is there a consensus about renewable energy? Is nuclear power still on the shareholders’ agenda? Is greenwashing profitable?

This research uses the event study methodology to analyze how the shareholders of leading US energy corporations (Thomson Reuters Top 100 Global Energy Leaders Ranking 2019) react to viral sustainability news. We downloaded all the viral news, from high-impact news published digitally (to carry out this study, we have downloaded the news from the databases Global Database of Events, Location, Language, and Tone (GDELT)), from January 2017 to December 2019.

We defined high-impact news with two standard deviations above the mean on the number of readings and media coverage indicator (indicator provided by GDELT). Ensuring that we only analyse the tail of the distribution; that is, the most relevant news.

The news was extracted from the Global Database GDELT and grouped by tone (GDELT Global Knowledge Graph (GKG) Version 2.0), type of energy, and environmental consequence, and each combination launched an event study.

The contributions of this study attempt to address multiple gaps we identified in the existing literature. First, this study extends the limited research concerning shareholders’ reactions to sustainability news. To the best of the authors’ knowledge and through searches in peer-reviewed databases, no previous study has empirically explored American energy shareholders.

Second, we improved earlier approaches because of the study’s level of detail and scale. By studying hundreds of thousands of news and segmenting them by tone and type of energy, we can thoroughly understand US shareholders’ preferences and biases.

Finally, the results of this study contribute to the existing literature by providing evidence of greenwashing incentives for US energy companies. For example, we identified positive news about fossil fuels impacting the stock market upwards significantly, while positive renewable energy news did not. Moreover, negative news impacted the stock market more negatively when analysing renewables than fossil fuels.

The remainder of the article is organized as follows:

The following section analyses the related literature, emphasising how investors value sustainability and news through stock market analysis. Thereafter, we discuss the sample and the methodology. Thirdly, the results and discussion section is presented and, finally, the conclusions.

### 1.1. Literature Review

This paper aims to understand the sustainability narrative of US energy companies’ shareholders through the news. We have chosen the news media as a source of information because the media has a crucial responsibility regarding variabilities in the stock market prices [26,27,28,29].

We consider three components to analyse the related work: news and the stock market, which discusses investors’ cognitive biases; secondly, sustainability and the stock market, which addresses shareholder reactions to sustainability information such as CSR reports or ESG ratings. Finally, we discuss sustainability news and the stock market, examining the existing literature and how this study fills the research gap in the literature.

#### 1.1.1. News and the Stock Market

Behavioural finance essays claim that shareholders are impacted by their cognitive bias and are unable to incorporate all the information and act in a rational way, in turn, overreacting to negative news [28,30,31]. Other researchers argued that the more media attention for certain companies, the higher trading volume or volatility [27,32,33,34,35].

Refs. [34,36,37] found that companies whose news was negative reported lower earnings. Similarly, other researchers have analysed how the press increasingly uses more emotional language to attract more attention, which affects the market to a greater extent [38,39]. Moreover, the scientific research suggests that the market is a mirror of the news, rather than a variable, and that it is the news that reacts to the market and not the other way around [29,40,41,42]. Finally, it is worth highlighting the finding of [43]. This Nobel laureate asserts that news can develop, such as a pandemic, and induce significant changes in the financial markets.

Based on the previous literature, we seek to understand whether the investors in our sample are negatively biased and whether the stock market becomes more volatile when few sustainability news becomes viral.

#### 1.1.2. Sustainability and the Stock Market

Some scientific investigations used the event study method to estimate the connection between sustainability and financial performance, albeit with a smaller sample size than in our study, and by handpicking. In general, these studies identified negative reactions from shareholders to negative environmental communication [14,44,45,46,47,48,49,50,51,52,53,54,55,56,57,58] and positive reactions to positive environmental communications [14,53,55,59,60,61,62,63].

Numerous studies have found positive relationships between sustainability and profitability outside of the event study methodology [24]. It detects an apparent correlation between sustainability and financial results in North America. They find that 75% of companies with sustainability reports and high ESG ratings reported better financial results than in the period when the ESG ratings were lower.

Moreover, [64] demonstrated the positive relationship between disseminating environmental information and profitability for companies that substantively address sustainability. Another work of critical research on the question is by [65]. They investigated 180 US companies for 18 years, finding that “high sustainability companies” outperformed “low sustainability companies”, both in the stock market as well as in terms of accounting performance.

Other studies that identify a positive relationship between CSR and sustainability include [66,67,68,69,70,71,72,73], amongst others.

#### 1.1.3. Sustainability News and the Stock Market

As discussed, shareholders use the news to remain informed, and their interest in ESG investment continues to grow. In addition, companies report ESG information, generally, once a year [74] in their ESG reports.

This research analyses sustainability news, as they possibly fill the gap in ESG information for the rest of the year. Shareholders can then evaluate the companies’ commitment to environmental activities and incorporate that information into their investment strategies.

Performing research concerning ESG news, [75] found that ESG news in China had a more significant impact on the stock market than unrelated ESG news, particularly environmental news.

Other recent studies regarding sustainability news found that companies with frequent positive news regarding sustainability have an excellent reputation, which protects them from financial losses in the stock market when negative news comes out, as the investor does not penalise them [7,14,58,76,77]. This tendency could indicate an incentive for greenwashing.

However, none of these articles compare news of different tones, and thus lose the nuance of the negativity bias. It remains necessary to analyse the energy sector responsible for the most CO_2_.

Nevertheless, similarly to the present study, [78] analyzed the reactions of European energy companies’ shareholders to sustainability news. They found that while sustainability news affected the stock market, there was no consensus among shareholders regarding renewable energy news. This indicates that the narrative was not homogeneous among the shareholders.

Based on these studies, we aim to understand whether the largest US energy companies react to sustainability news and the difference between the US and European markets, as the European continent doubles the percentage of sustainability assets traded compared to the US [23]; possibly indicating a more outstanding commitment from its shareholders.

## 2. Materials and Methods

### 2.1. Materials

For this study, we downloaded the publication dates of all relevant news posted digitally, globally, concerning sustainability and the leading American energy firms. We also compiled the closing prices of the American stock markets where the companies in our sample are listed. Table 1 presents the data sources.

GDELT is a major news repository. It is open-source and displays all the news published online, across the globe, in 100 different languages. Ref. [79] described it as a tale of the world.

For each news article, GDELT offers the publication date, its “news volume” (combination its coverage and of the number of times some news is read) and segmentation by tone: negative, neutral, and positive. These segmentations were performed using natural language processing techniques (GDELT Global Knowledge Graph (GKG) Version 2.0).

For the tone of news, GDELT uses 51 data dictionaries, including the following widespread dictionaries: “Harvard IV-4 Psychosocial Dictionary” “Harvard IV-4 Psychosocial Dictionary” [80], the “WordNet-Affect dictionary” [81,82], the “Loughran and McDonald Sentiment Word Lists dictionary” [83,84].

GDELT also offers the possibility of combining all the news that inform the same theme on one date, using the date with the highest news volume. We have used this option to avoid repeating events in our event studies.

Our search method in GDELT—devised using Python programming language—is based on a combination of three terms: company name, type of energy, and environmental consequence, and produced 12,172 combinations. Table 2 presents the terms.

The environmental consequences keywords in this article have been selected according to the planetary boundaries defined by [85]. Therefore, we used their defined planetary boundaries and their causes and consequences.

From each of the word combinations, we downloaded all the news whose news volume (the combination of the number of times the news is read and its coverage, by GDELT) was more than two standard deviations from the average of all the news. This ensures that we analyse only the tail of the distribution, focusing on the news with the most significant impact. This resulted in 207,386 high-volume news about sustainability.

We downloaded data on the date of the news, news volume, and tone from GDELT.

### 2.2. Methods

We applied the event study methodology outlined in [86] to conduct this study. This event study methodology has become a standard method to measure the reaction of share prices to an advertisement or event, since [87,88] introduced it. The methodological assumptions of this research, common in the event study methodology, are that the financial markets’ efficiency is semi-strong and the stock prices should immediately reflect the news information [89]. Second, the assumption is that the event is unexpected and, finally, the non-occurrence of other events during the event window is also assumed.

To examine, in depth, all the news downloaded from GDELT, we grouped them by tone, energy, and environmental consequence keyword (for example, Positive Tone, Fossil fuel, CO_2_), carrying out five event studies for each of the 1485 combinations.

We analyzed the cumulative average abnormal returns (CAAR) and abnormal volatilities (AV). In the case of CAAR, we used 1485 combinations per five expected return models, resulting in 7425 CAAR event studies and 1485 Avs event studies; as for AV, we performed one market model.

As the first step of the event study, the day of interest and the period over which the stock prices will be studied (i.e., the event window) must be defined.

- Day of the event: The day of the event is the date when the news with the highest volume intensity was published. We wrote the code so that news with the same content as the peak news was not downloaded seven days before and seven days after to guarantee the event window.
- Event window: (−7, 7). Following the research of [43] on Narrative Economics, we assumed that viral, high-volume news spread in a similar fashion to an epidemic curve, with published news before and after the news with the highest peak. Therefore, we used CAARS to understand the total effect of the spread rather than just the peak. The news spread could be different depending on the type of news. However, we assumed an event window of 14 days would catch the effect, as [90] argued that one advantage of averaging the results is that the law of large numbers offsets the errors of having very long or small windows.

The study was conducted based on events between 2017 and 2019. The event window was defined as the period of seven days before the event and seven days after the event. Day zero denotes the day of publication of the news. The estimation window, used to predict normal returns, was defined as the period corresponding to 99 days before the event window. Logarithmic returns were used in both windows.

Assessing the impact of the event requires a measure of abnormal performance (AR). Five expected return models were used to model the AR; as there is no consensus in the scientific literature about which expected return model is best, with one side arguing that the market model gives results as good as other complex models [91,92], and the other side claiming that the market models has serious doubts [93,94], we looked for the consensus of at least three of the five expected return models to validate our results and reduce potential errors.

The models used are as follows: market model (mm), market-adjusted model (mam), comparison period mean-adjusted (cpmam), generalised autoregressive conditional heteroscedasticity (GARCH), and exponential generalised autoregressive conditional heteroscedasticity (EGARCH) models.

- The (mm) is commonly used for event study analysis. This model considers the actual returns of a baseline reference market and tracks the correlation of a company’s stock with the baseline. Equations (1) and (2) specify the model. The abnormal return on a particular day, *AR_it_*, in the event window describes the difference between the actual stock return, *R_it_*, on day *t*, and the expected return, which is foretold based on two facts; the average relationship between the firm’s stock and its reference market (expressed by the *α* and *β* parameters), and the actual reference market’s return, *R_mt_*.
  (1) $$R_{it}=\alpha_{i}+\beta_{i}\cdot R_{mt}+\varepsilon_{it}$$
  then
  (2) $${AR}_{it}=R_{it}-\left(\alpha_{i}+\beta_{i}\cdot R_{mt}\right)$$
- (mam) is used to handle the event’s potential consequences in the stock market. In the (mam), the followed return of the reference market on day *t* (*R_mt_*) is extracted from the return *R_it_* for the observation *i* on day *t*. Equation (3) establishes the *AR_it_*:
(3) $$AR_{it}=R_{it}-R_{mt}$$
- In the (cpmam), the abnormal return in the event window is the return of observation *i* on day *t* minus the average return of the observation *i* in the estimation window (Equations (4) and (5)):
  (4) $$AR_{it}=R_{it}-\bar{R_{i}}$$
  where
  (5) $$\bar{R_{i}}=\frac{1}{T_{1}-T_{0}}\;\underset{t\epsilon \left(T_{1},T_{0}\right)}{\sum }R_{it}.$$
- The (GARCH) uses a market model single factor with GARCH (1, 1) errors estimated, particularly:
(6) $$R_{it}=c_{i}+\beta_{i}R_{mt}+\gamma_{i}D_{it}+\varepsilon_{it},$$

The conditional variance [95] may be written as:

(7) $$\sigma_{it}^{2}=\alpha_{i0}+\alpha_{i1}\epsilon_{i\left(t-1\right)}^{2}+\lambda_{i}\sigma_{i\left(t-1\right)}^{2}+\delta_{i}D_{it}.$$

where *D_it_* is a dummy variable which takes 1 on the disclosure day *t* and 0 otherwise for firm *i*; and *ε_it_* are the volatility and the errors of firm *i*, respectively. In addition, *R_it_* is the return of firm i and *R_mt_* is the return of the reference market *m*, both on day *t*. Equations (6) and (7) represent the mean and time-varying volatility functions, respectively. The abnormal returns and abnormal volatility caused by the publication of sustainability news are measured by *γ* and *δ_i_* for firm *i*. Parameters are estimated by maximum likelihood (a non-linear solver is used for the optimization problem).

- The (EGARCH), Ref. [96] proposed the EGARCH model to include the asymmetric effect of changes in the prices of an asset on its volatility. The Garch (1, 1) model does not account for any asymmetry that may arise from the negative and positive moves of the market or as it is usually called, the leverage effect. To solve this, the EGARCH model applies a logarithmic conditional variance. Equation (8) is the conditional variance of the EGARCH (1, 1) model:
  (8) $$\ln(\sigma_{it}^{2})=\omega_{i}+\beta_{i}\ln(\sigma_{i\left(t-1\right)}^{2})+\alpha_{i}\left|\frac{\varepsilon_{i\left(t-1\right)}}{\sigma_{i\left(t-1\right)}}\right|+\gamma_{i}\frac{\varepsilon_{i\left(t-1\right)}}{\alpha_{i\left(t-1\right)}}.$$
  where *ω* corresponds to a constant, *β* is the now logarithmic GARCH term, *α* is the ARCH term that no longer has to be positive. The *γ* is the so-called leverage term; if is significant and different from zero there will be asymmetry in the estimation period. The *σ* is the standard deviation.

The expected returns have been obtained from the estimated coefficients for each firm and market. We used a pre-event period that starts on day −110 and finishes on −11, day 0 being the day of publication of the analysed new.

Cumulative abnormal returns (*CAR_it_*), refers to the sum of abnormal returns (*AR_it_*) over a given period of time, the event window.

(9) $$CAR_{it}=\overset{t2}{\underset{t=t1}{\sum }}AR_{i}$$

Average Abnormal Returns (*AAR_t_*) aggregates the abnormal returns (*AR_it_*) for all *n* stocks to find the average abnormal return at each time *t*.

(10) $$AAR_{t}=\frac{1}{n}\overset{n}{\underset{i=1}{\sum }}AR_{it}$$

Cumulative Average Abnormal Return (*CAAR_t_*), Equation (10), sums the *AAR_it_* for the event window.

(11) $$CAAR_{t}=\overset{t2}{\underset{t=t1}{\sum }}AAR_{t}$$

to test our hypothesis, the parametric skewness-adjusted *t*-test was used, [97].

In the case of this investigation, the returns were skewed as we performed event studies that were segmented by tone: the negative and neutral news items were negatively skewed, and the positive items were positively skewed.

The authors of [98,99,100] found that the skewness-adjusted *t*-test that was introduced by [97] performed as well as equivalent non-parametric tests, as long as the sample size was not small.

Recalling the (unbiased) cross-sectional sample variance as:

(12) $$\sigma \left(CAAR_{t}\right)=\sqrt{\frac{1}{n-1}\overset{n}{\underset{i=1}{\sum }}{\left(CAR_{it}-CAAR_{t}\right)}^{2}}$$

Then the skewness estimation focused on averaged abnormal returns is specified by:

(13) $$t_{skew}=\sqrt{n}\left(S+\frac{1}{3}\hat{\gamma }S^{2}+\frac{1}{6n}\hat{\gamma }\right),$$

(14) $$\hat{\gamma }=\frac{\sum_{i=1}^{n}(CAR_{it}-CAAR_{t)}}{n\sigma (CAAR_{t)}}$$

where

(15) $$S=\frac{CAAR_{t}}{\sigma \left(CAAR_{t}\right)}$$

where $\hat{\gamma \;}$ is the estimate of the coefficient of skewness and is the skewness-adjusted *t*-test.

Once the abnormal returns are calculated, it is necessary to determine if the deviation from the normal return is a statistically significant. To achieve this, a standard t-test is applied with the hypothesis test defined as:

The abnormal returns cannot be distinguished from zero

H0: *CAAR* = zero

The abnormal returns can be distinguished from zero

H1: *CAAR* ≠ zero

The decision is to reject H0 if *t_skew_* > tcritical or *p*-value < 0.1. This means that the value is statistically significantly different from zero, with a significance level of 5%.

This means that if the tskew is greater than 1.96 or minor than −1.96, we reject it. If we do not reject it, because the *t_skew_* is less than 1.96, this indicates that the results are not statistically different from zero.

The initial number of event studies was 7425; following [101], we discarded all the studies with less than 50 news to ensure a sufficient sample size to guarantee robust results.

The companies in our sample may have conflicts of interest with each other as the core business of some are fossil fuels, while others are in renewable energies; thus, we also analyzed abnormal volatilities (AV). When analyzing the returns in absolute values, we can observe reactions that were not detected by *CAAR* because they were compensating each other.

A single-day test statistic was performed [102]. The time series is utilised as a whole, and thus, there is no need for an event window. The market model with GARCH errors was used, based on Equations (6) and (7).

We calculated another cross-sectional t-statistic to test whether the conditional volatility on announcement day is different from the other days across the firms. We used the parametric test: average of cross-sectional-corrected-vy *t*-test. This statistic standardises by the standard deviation of firm *i* during the entire period:

(16) $$t(\delta_{i})=\left\{\sum_{i=1}^{n}S_{i}/n\right\}/{\left\{\left[1/n(n-1\right]\overset{n}{\underset{i=1}{\sum }}{\left[S_{i}-\overset{n}{\underset{j=1}{\sum }}S_{j}/n\right]}^{2}\right\}}^{0.5}$$

where $S_{i}=\delta_{i}/{\hat{\delta }}_{i,0}$ represents the adjustment of $\;\delta_{i}$ by the estimated volatility of firm *i* on the day of the publication of the sustainability news.

As with CAARS, all analyses with less than 50 news were discarded.

To test if the AV were statistically significant, we used the Cross-Sectional-Corrected-Vy-t-Test with a significance level of 5%, as performed with the CAARs.

All the event studies performed in this research used the ‘EventStudy’ package by [103], hosted on RapidAPI and executed in Python.

## 3. Results and Discussion

### 3.1. Results

In the result section, we will discuss first the *CAAR* results and then the AV.

#### 3.1.1. Cumulative Average Abnormal Returns (CAAR)

**Figure 1:**
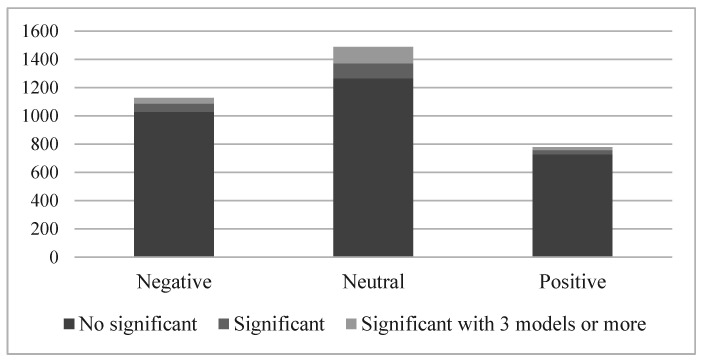
*CAAR* Event studies results summary by tone.

Figure 1 illustrates the outcomes of the 3393 studies performed, discerning their statistical significance and the consensus of the models. We aggregated the event studies results by tone.

Figure 1 shows that most of the news did not provoke a reaction among the shareholders, and only 11% did, being statistically significant. If, in addition, we demand consensus of three or more expected return models, only 5% of the news in our sample impacted shareholders’ decision-making. Regarding the news tone, the analysed news had a clear negative bias, as negative and neutral news were higher in number than positive, representing 73% of the sample.

Similarly, the highest proportion of significant news was for neutral news, with 15% of news affecting investor investment movements, followed by negative (9%) and positive (6.5%) news. These results confirm the assumptions of [28] that investors react more to negative news and that news media more often cover negative than positive news.

Table 3 aggregates the results of all significant event studies as confirmed by three or more expected return models. Based on Table 3, we conclude that negative and neutral news caused negative statistical significance reactions, which implies a drop in the share’s prices. Meanwhile, the positive news had no statistically significant aggregated results, which indicates that the positive news event studies were compensating each other; in other words, there is no consensus among the shareholders.

These results align with the negativity bias that confirms that humans tend to pay more attention to negative than positive experiences or outcomes, by a ratio of four to one [104,105]. Thus, negativity bias could also influence shareholders’ investment judgments.

Table 4 aggregates the results seen in Figure 1 by type of energy. We can observe that the most abundant news and the highest proportion of significant news corresponded to that of renewable energies, especially those with a neutral tone, as 17% of these event studies were significant. In contrast, news about nuclear energy was less frequent and less significant.

Table 5 reports the aggregated results for all event studies by type of energy and news tone. The main conclusion from Table 5 is that negative news, regardless of the energy type, caused statistically significant negative reactions, thus confirming the negativity bias. Similar results were observed for the neutral news. In the same way as with negative news, all reactions to neutral news were statistically significant and negative.

Both positive and negative news on nuclear energy did not react to the companies’ prices in our sample, although the neutral news did.

Notably, there was a non-statistically significant reaction found for positive news about renewable energy, indicating a slight lack of consensus among shareholders on the sustainable energy narrative. Finally, these results highlight that the news about fossil fuels was the one that had the most significant repercussion in terms of statistical significance. This news received a reaction from the market across all news tones, and hence, it could be said that fossil fuels are still the main engine of investor attention.

The key conclusion that we can obtain from the *CAAR* analysis is that there was a clear negativity bias among shareholders in the face of sustainability news, which extended to news of a neutral tone. The news about renewable energy was the most frequent and with the highest percentage of significant event study results, indicating a clear interest in renewables among the shareholders. However, their narrative was not shared for positive news, suggesting that shareholders’ interests continued to be in fossil fuels. Consequently, they might see renewables as a threat.

#### 3.1.2. Abnormal Volatilities (AV)

We conducted a study to check if the *CAAR* analysis was able to detect all shareholder reactions. When working with absolute values, the AVs detect movements in the stock market that the CAARs cannot find since they are offset. Thus, statistically significant AV results will indicate that the assets analyzed have undergone significant variations in their prices—the greater the volatility, the greater the risk and the potential losses for shareholders.

**Figure 2:**
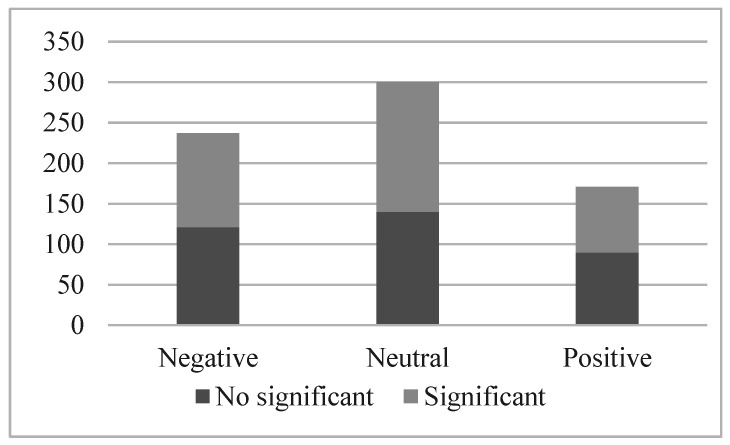
AV Event studies results summary by tone.

Figure 2, similar to Figure 1, shows us the number of event studies that are statistically significant. However, contrary to Figure 1, when we analyze volatilities, we find that around half of the event studies carried out are significant, regardless of the tone. These AV reactions would indicate that although in terms of *CAAR*, no response was observed from shareholders for most of the news, the AVs reveal that the market becomes more volatile in the face of sustainability news.

Table 6 aggregates the results of all the AV event studies by the tone of the news, and all were found to be statistically significant. In Table 3, where we analyzed the same parameters using CAARs, we found a non-significance of the positive news. The AV reaction to positive news confirms that there is no consensus on the sustainability narrative among shareholders. What is considered good news in terms of sustainability would raise the stock price for some, while others may consider it to be negative news. Therefore, no *CAAR* reaction was found because the effects offset each other.

Table 7 shows the number of statistically significant event studies by energy. The main conclusion from this table is that the news about renewables was the most abundant, but this did not translate into a greater reaction from the stock market. The news items with the highest proportion of significant event studies were fossil fuels, followed by news on nuclear energy. Notably, while the news on nuclear energy was rare, most of these events made the market more volatile.

Finally, Table 8 presents the results of all the statistically significant AV event studies, aggregated by type of energy and tone of the news. As in Table 5, all the event studies analyzed were significant. This again confirms that shareholders follow sustainability news and make investment decisions based on it, making the stock market more volatile.

### 3.2. Discussion

Fossil fuels continue to have a predominant position in the American market, wherein shareholders are affected more by sustainability news and exhibit expected behaviour, with prices dropping for negative news and vice versa for positive news. While the highest number of news data was about renewable energy, the shareholders reacted less significantly, showing a non-consensus for the sustainability narrative. This lack of consensus is visible in Table 5, where positive news about renewables was not statistically significant; however, the positive news on renewables was significant when we analyzed AV (Table 8).

These results are similar to those found by [78] on European energy companies. The main difference is that in the case of Europe, the news on renewable energies found CAARs of the opposite sign to the tone of the analyzed news, indicating that the shareholders’ interests were still highly linked to fossil fuels and saw renewable energies as a threatening competition. However, the US shareholders do not react to them.

Both studies show that the shareholders’ interests remain in fossil fuels energy. Fossil fuel companies have a more profitable business case with positive news about their operations than renewable energy companies, thus making a case for greenwashing.

Policymakers should consider these insights because if there are no economic or legal incentives to change the shareholders’ sustainability narrative in the short term, shareholders will focus on profit maximization and not act out of ecological awareness. [106] argued that a carbon tax is a cost-effective policy to change the narrative and reduce the U.S.’ GHG emissions, as it has happened in 23 countries and even with a growth in the GDP in places such as British Columbia.

The findings presented and discussed in this study are based on the US and Europe, and we cannot ensure that other country’s shareholders would act in the same way.

## 4. Conclusions

This study aimed to understand the sustainability narrative of the shareholders in energy companies in the US to elucidate if the investors value the companies’ efforts to be sustainable or if they continue to reward business as usual to guarantee profitability.

To achieve this, we analysed all the news on sustainability, worldwide, in reference to the leading American energy companies, from 2017 to 2019, that included a combination of keywords in their articles (Table 1), including the analysed companies, energy, and environmental consequences. Moreover, we segmented all the news by tone: negative, neutral, and positive. We downloaded 207386 news items from GDELT and carried out 7425 event studies, analyzing CAARs and AVs.

By analysing the news, conducting thousands of event studies, and segmenting them by energy type and tone of the news, we were able to identify the biases of the US energy shareholders. Our contribution to existing literature is to affirm that all shareholders do not share the sustainability narrative and that fossil energies continue to be rewarded, indicating a clear incentive for greenwashing. We also confirm the negativity bias.

After analysing the CAARs and AVs, we can draw several conclusions. The main conclusion is that negative and neutral news were the most abundant and influenced the stock market downside, showing a clear negative bias. Furthermore, this negative bias was observed for all types of energy. The results also highlight that the news on renewables was the most frequent, with the most significant event studies; however, the analysis using CAARs revealed how the results on positive news are offset, indicating a non-consensus of shareholders, which in turn was supported by the AV analysis. Ultimately, we identified that positive news about fossil fuels positively impacts the market to a greater extent than positive renewable energy news. This provides the empirical evidence for the practice of greenwashing by businesses.

This research faced the classic limitation of the event study methodology. Primarily, the CAARs cannot exclusively be the consequence of the sustainability news. Subsequently, determining a precise estimation period is problematic; with long windows, while the confounding effect with other events can occur, there is a possibility of not catching the real media repercussion with shorter windows. Moreover, the data sources could have omitted keywords to make the news search more accurate.

Future directions of this study should focus exclusively on renewable energy companies to analyse whether shareholders interested in renewables have a standard sustainability narrative and overreact to negative news. It would also be very enriching to the current literature to compare the reactions of the shareholders of the countries with current carbon taxes and those without, to see if their narrative towards sustainability changes and makes them more demanding. Finally, another line of research could focus on the event study methodology using the results of this research to analyse which expected return model behaves more adequately and accept or reject the hypothesis that the market model behaves as good as the complex ones [91,92].

## Figures and Tables

**Table 1:** Data sources United States.

Companies	North American companies included in the Thomson Reuters Top 100 Global Energy Leaders Ranking 2019
News	GDELT (The Global Database of Events, Language, and Tone), package: GDELT Global Knowledge Graph (GKG) Version 2.0
Stock data	Yahoo Finance

**Table 2:** News Selection criteria.

USA Companies	Energy	Environmental Consequences Keywords
Anadarko	Gas	Nitrogen
Marathon oil corp	Fossil Fuels	Phosphorus
Avangrid	Renewables	Carbon dioxide
Chevron corp	Nuclear	CO_2_
CMS Energy	Coal	Methane
ConocoPhillips	Solar	Ozone
ExxonMobil	Hydro	Pollution
Covia Holding	Wind	Waste
First Solar	Biomass	Plastic
Halliburton Comp	Geothermal	Footprint
Hess Corp	Marine	Aerosol
Marathon Oil Corp	Tidal	Global warming
Ni Source	Petrochemical	Emissions
Occidental Petroelum Corp	Petrol	Greenhouse gas
Philips 66	Petroleum	Air quality
Schlumberger	Ethanol	Sea level
Sempra Energy		Climate change
Sun Power		Extreme weather
Vestas		Natural resources
Enel		Biodiversity
		Toxic
		Extinction
		Nitrogen cycle
		Ocean acidification
		Land use
		Fresh water
		Depletion
		Chemical Pollution
		Overexploitation
		Sustainability
		Ecosystem

**Table 3:** CAAR results from all statistically significant event studies with consensus aggregated by tone.

Tone	Average of Skewness Corrected T	Average of *CAAR* Value	Average of *p* Value
Negative	−2.4517 *	−0.0168	0.0240
Neutral	−2.2449 *	−0.0142	0.0175
Positive	−0.0207	0.0001	0.0255

* Statistically significant.

**Table 4:** Number of statistically CAAR significant event studies with consensus by type of energy.

Energy	Negative		Neutral		Positive	
	No Statistical Significance	Statistical Significance	No Statistical Significance	Statistical Significance	No Statistical Significance	Statistical Significance
Fossil fuels	425	26	468	71	319	17
Renewables	488	69	689	141	353	30
Nuclear	115	4	109	10	55	4

**Table 5:** CAAR results from all statistically significant event studies with consensus by tone and energy.

Energy	Negative			Neutral			Positive		
	Average of Skewness Corrected T	Average of *CAAR* Value	Average of *p* Value	Average of Skewness Corrected T	Average of *CAAR* Value	Average of *p* Value	Average of Skewness Corrected T	Average of *CAAR* Value	Average of *p* Value
Renewables	−2.4699 *	−0.0172	0.0221	−2.4483 *	−0.0152	0.0156	0.0236	0.0006	0.0222
Fossil fuels	−2.3697 *	−0.0151	0.0293	−1.7788	−0.0117	0.0177	2.1176 *	0.0114	0.0371
Nuclear				−2.4968 *	−0.0132	0.0198			

* Statistically significant.

**Table 6:** AV results from all statistically significant event studies aggregated by tone.

Tone	Average of Cross-Sectional-Corrected-Vy-*t*-Test	Average of *p* Value
Negative	2.5984 *	0.0181
Neutral	2.4758 *	0.0206
Positive	2.5231 *	0.0200

* Statistically significant.

**Table 7:** Number of statistically significant AV event studies by type of energy.

Type of Energy	Negative		Neutral		Positive	
	No Statistical Significance	Statistical Significance	No Statistical Significance	Statistical Significance	No Statistical Significance	Statistical Significance
Fossil fuels	39	52	51	57	32	42
Renewables	72	50	81	87	51	34
Nuclear	10	14	8	16	7	5

**Table 8:** AV results from all statistically significant event studies by tone and energy.

Type of Energy	Negative		Neutral		Positive	
	Average of Cross-Sectional-Corrected-Vy-*t*-Test	Average of *p* Value	Average of Cross-Sectional-Corrected-Vy-*t*-Test	Average of *p* Value	Average of Cross-Sectional-Corrected-Vy-*t*-Test	Average of *p* Value
Renewables	2.4264 *	0.0217	2.3692 *	0.0237	2.3526 *	0.0272
Fossil fuels	2.6500 *	0.0188	2.5443 *	0.0191	2.5156 *	0.0214
Nuclear	2.4961 *	0.0175	2.5598 *	0.0179	2.3002 *	0.0270

* Statistically significant.

## Data Availability

Publicly available datasets were analysed in this study. These data can be found at: https://www.gdeltproject.org/globaldashboard/ (accessed on 12 February 2021) and https://Yahoofinance.com (accessed on 26 March 2021).
